# Guilt and Unfinished Business in Bereavement: Rumination as a Pathway to Prolonged Grief and Trauma

**DOI:** 10.3390/jcm14238582

**Published:** 2025-12-03

**Authors:** Sara Albuquerque, Beatriz Barbas Pereira, Alexandra Coelho, Ricardo Pinto

**Affiliations:** 1 HEI-Lab: Digital Human-Environment Interaction Labs, Lusófona University, Campo Grande 376, 1749-024 Lisboa, Portugal; ricardo.pinto@ulusofona.pt; 2 Academia Transformar, Av. António Augusto de Aguiar 17 5th Floor, 1050-012 Lisboa, Portugal; beatrizbarbaspereira@gmail.com; 3 ISPA—Instituto Universitário de Ciências Psicológicas, Sociais e da Vida, R. do Jardim do Tabaco 34, 1149-041 Lisboa, Portugal; alexandra.moura.coelho@gmail.com

**Keywords:** bereavement, prolonged grief, rumination, cognition, guilt, unfinished business, ptsd, psychological distress, mediation, longitudinal studies

## Abstract

**Background**: Bereavement often elicits complex emotional and cognitive reactions, including guilt, unresolved relational concerns, and repetitive negative thinking. Although grief-specific rumination is recognized as a key process that can intensify prolonged grief and trauma-related responses, longitudinal evidence clarifying its mediating role remains limited. This study examines whether grief-specific rumination explains how guilt and unfinished business contribute to prolonged grief and PTSD symptoms over time. **Methods**: A two-wave longitudinal design was used to assess bereaved adults several months after loss and again at follow-up. Measures included guilt, unfinished business, grief-specific rumination, prolonged grief symptoms, and PTSD symptoms. Mediation analyses tested whether rumination accounted for associations between early loss-related responses (guilt and unfinished business) and psychological outcomes at follow-up. **Results**: Grief-specific rumination consistently emerged as the mechanism linking guilt and unfinished business with both prolonged grief and PTSD symptoms. These findings suggest that the impact of these early loss-related responses operates primarily through persistent and repetitive cognitive engagement with the loss. **Conclusions**: Rumination emerged as a central mechanism through which guilt and unfinished business shape bereavement-related distress. These findings highlight the importance of interventions that directly target repetitive negative thinking to promote healthier adjustment following loss.

## 1. Introduction

Bereavement is a universal experience that can profoundly affect psychological, social, and physical well-being [1]. Although most individuals adapt to loss over time, a significant minority develop persistent difficulties that reflect disruptions in emotional and cognitive processing [2,3]. Recent work highlights the relevance of specific cognitive and emotional factors—such as guilt, unresolved relational issues, and repetitive negative thinking—in shaping bereavement trajectories [4,5,6]. Nonetheless, the way these factors jointly contribute to maladaptive outcomes—particularly over time—remains insufficiently understood, underscoring the need for longitudinal research.

The grieving process is a multifaceted experience that involves physical, psychological, behavioral, spiritual, and sociocultural dimensions, and is commonly marked by emotions such as guilt, anger, remorse, regret, shame, and persistent [7]. For most bereaved individuals, the intensity of these reactions decreases over time, allowing for gradual adaptation to the loss [3]. However, a significant minority—around 10%—experience enduring and maladaptive responses that severely impair functioning [2,8].

The recognition of these enduring responses has led to the formal inclusion of Prolonged Grief Disorder (PGD) in both the DSM-5-TR [9] and the ICD-11 [10]. PGD is characterized by a persistent preoccupation with the deceased, intense emotional pain, diminished capacity to participate in social or pleasurable activities, difficulty accepting the reality of the death, and significant impairment in daily life.

The experience of loss can also operate as a traumatic event with profound psychological and physical consequences [11]. Although PGD and post-traumatic stress disorder (PTSD) are recognized as distinct syndromes, empirical studies highlight their overlapping features, such as intrusive memories, hyperarousal, and disturbances in emotional regulation [12]. Other responses frequently include disrupted sleep, concentration difficulties, persistent anxiety, exaggerated startle responses, and feelings of emptiness or hopelessness, combined with a breakdown of basic assumptions about safety, trust, and control [13,14]. Such reactions are particularly pronounced after sudden or violent deaths, when the loss not only elicits profound emotional pain but also destabilizes core beliefs about predictability and security. Importantly, substantial distress may also arise in the context of prolonged anticipatory grief [13,15].

Among the emotional responses that can shape these trajectories, guilt stands out as especially salient. Being one of the most commonly reported experiences in bereavement [4], guilt is explicitly recognized among the diagnostic features of PGD in the DSM-5-TR [9]. Feelings of guilt may arise from perceptions of having failed in caregiving, unresolved conflicts, or thoughts that the death could have been prevented or the deceased treated better [16]. Prevalence estimates vary widely, with systematic reviews reporting rates between 30% and 60% among bereaved individuals [4], reflecting its crucial role in shaping the trajectory of bereavement.

Theoretical perspectives differentiate guilt from shame: while both are self-conscious emotions requiring self-awareness, guilt is oriented toward specific behaviors and often elicits reparative tendencies, whereas shame targets the global self and is more strongly linked to feelings of worthlessness and avoidance [17,18,19]. Importantly, guilt may therefore function in both adaptive and maladaptive ways. It may foster moral awareness, empathy, prosocial behavior, and even concern for the deceased when adaptive [20,21], yet associated with depression, obsessive-compulsive symptoms, PGD, and PTSD when maladaptive [16,22].

Closely connected to guilt is the concept of unfinished business, defined as unresolved relational issues, unexpressed feelings, or perceived lack of closure with the deceased [23,24]. Examples include unresolved conflicts, the desire to apologize, to express gratitude, or to declare love [25]. These experiences are relatively common, with nearly half of bereaved individuals reporting them [24]. Although frequent, unfinished business is consistently identified as a risk factor for PGD [23] and is often accompanied by emotions such as anger, remorse, and guilt [26]. Higher levels of unfinished business, missed opportunities, or unresolved relational conflicts are associated with more severe grief symptoms [7]. Moreover, empirical studies suggest that guilt may mediate the relationship between unfinished business and prolonged grief [7], highlighting the interconnectedness of these processes.

A cognitive mechanism that could potentially explain the link between guilt and unfinished business to maladaptive outcomes is rumination. Defined as repetitive and uncontrollable thinking about one’s emotions and problems without generating constructive solutions [27], rumination is considered a transdiagnostic risk factor across psychopathologies. In the context of bereavement, interest has shifted from depressive rumination to grief-specific rumination, which centers on the irreversibility of the loss and its relational and existential consequences [28]. It often takes the form of counterfactual thinking, persistent questioning about the causes and consequences of the death, reflections on its unfairness, and preoccupation with the meaning of the loss [5,6,28]. Research consistently shows that grief rumination predicts PGD more strongly than depressive rumination and is also linked to depressive, anxious, and post-traumatic symptoms [5,6].

The two most systematically investigated—and somewhat competing—theoretical models of rumination in the context of bereavement are The Responses Styles Theory (RST) and the Rumination as Avoidance Hypothesis (RAH) [6]. The RST posits that rumination exacerbates grief by amplifying negative cognitions, impairing problem solving, reducing engagement in restorative activities, and undermining social support [29]. In contrast, the RAH views it as a strategy to avoid confronting the permanence of loss, which ultimately prevents integration of the loss into autobiographical memory and impeding acceptance [1,30]. Despite their differences, both models converge on the view that maladaptive rumination perpetuates distress, whereas more adaptive reflective forms may contribute to meaning-making and adjustment [5].

Rumination has also been identified as a mediating mechanism linking negative emotions to psychopathological outcomes with early studies demonstrating its role in the relationship between self-blame and depressive symptoms [31,32]. Likewise, trauma models have proposed that appraisals such as guilt trigger maladaptive strategies, especially repetitive negative thinking, that intensify post-traumatic symptoms [33].

Given that both guilt and unfinished business are prominently present in grief trajectories [4,24], it is theoretically plausible that rumination may mediate the associations between these factors and grief and trauma symptoms. Nevertheless, empirical investigations specifically addressing these relationships remain scarce. Wang et al. [34], for instance, demonstrated that rumination mediates the link between guilt and the development of trauma-related reactions in bereaved individuals. In a similar vein, cognitive models of prolonged grief disorder (PGD) propose that negative appraisals of the loss play a central role in maintaining symptoms [11,35]. However, to our knowledge, no studies have explicitly examined rumination as a mediator connecting guilt and unfinished business with both PGD and posttraumatic stress symptoms, leaving this area of research underdeveloped.

The present study seeks to address this gap by testing whether rumination mediates the longitudinal association between guilt, unfinished business, and the development of PGD and PTSD symptoms. By clarifying this mechanism, the study contributes to a deeper understanding of cognitive pathways in maladaptive grief and offers empirical support for clinical interventions that target rumination to alleviate bereavement-related distress.

The following hypotheses were formulated:

**H1:**  *Higher levels of guilt are positively associated with greater symptoms of prolonged grief and PTSD.*

**H2:**  *Greater perception of unfinished business with the deceased is positively associated with greater symptoms of prolonged grief and PTSD.*

**H3:**  *Rumination mediates the relationship between guilt and symptoms of prolonged grief and PTSD.*

**H4:**  *Rumination mediates the relationship between unfinished business and symptoms of prolonged grief and PTSD.*

## 2. Method

### 2.1. Study Design

This study employed a quantitative longitudinal design to examine how grief-related psychological processes evolve over time following the death of a loved one. Longitudinal designs are considered particularly valuable for studying dynamic psychological responses to loss, as they enable the examination of temporal patterns and potential causal mechanisms with greater precision than cross-sectional approaches [36,37]. When evaluating mediation models specifically longitudinal designs are particularly relevant since this statistical method infers causality [38].

Data were collected at two time points—between three and six months post-loss and again between nine and twelve months—to capture change in key variables such as prolonged grief symptoms, PTSD symptoms, guilt, unfinished business, and rumination. Guilt and unfinished business were assessed only at Time 1 (T1), whereas rumination, grief symptoms, and posttraumatic stress symptoms were assessed at both T1 and Time 2 (T2). This design therefore provides temporal precedence for the hypothesized paths, with predictors measured exclusively at T1 and outcomes re-assessed at T2. Only participants who completed both T1 and T2 assessments were included in the analyses, resulting in a final matched longitudinal sample with no attrition between waves. This design permitted a more robust examination of how these variables unfold over time and relate to the construction of meaning following loss.

The sample consisted of adults (≥18 years) who had experienced the death of a loved one during the COVID-19 pandemic (, regardless of the cause of death. To avoid including individuals in the acute mourning phase, only participants whose loss had occurred at least three months prior were eligible. A non-probabilistic snowball sampling method was used, whereby participants could share the questionnaire link with others meeting the inclusion criteria.

The sample comprised 141 Portuguese participants, predominantly female (87.9%), with ages ranging from 18 to 76 years (*M* = 42.38; *SD* = 12.16). Most were married or living with a partner (61%), had completed higher education (61.9%), and were employed full-time (70.9%). Concerning the loss, 73% reported an extremely close relationship with the deceased, most frequently a parent (50.4%), followed by spouse/partner (7.8%) or child (7.1%). Cancer was the leading cause of death (41.8%), followed by organ failure (26.2%) and sudden death (13.5%); one participant (0.7%) reported suicide.

### 2.2. Procedure

Data collection was conducted online via a secure link between late 2020 and mid-2021. The questionnaire began with informed consent, which described the study objectives, inclusion criteria, voluntary nature of participation, potential emotional risks (e.g., evocation of painful memories, intrusive thoughts, or sadness), and the right to withdraw at any time. Participants willing to take part in the second wave of data collection were invited to provide their contact details.

Several ethical safeguards were implemented to protect participants’ emotional well-being. Data collection was temporarily suspended during festive periods, which may exacerbate emotional vulnerability. To minimize potential distress, sociodemographic questions and other items deemed less emotionally activating were placed at the end of the questionnaire. In addition, participants were provided with information on psychological support resources, including national health hotlines, specialized psychological support lines, and mental health services in their area of residence, should they require additional assistance.

The study was approved by the Ethics Committee of regional health departments and was conducted in accordance with the Declaration of Helsinki.

### 2.3. Measures

Sociodemographic and loss-related questionnaire. Participants first completed a brief questionnaire assessing sociodemographic characteristics (e.g., age, gender, nationality, professional status) and information related to the death (e.g., degree of kinship, cause of death, and place of death).

Impact of Event Scale—6 (IES-6; Norway [39]; Portuguese version [40]). The IES-6 is a six-item self-report scale designed to assess distress related to traumatic or stressful events. Items are rated on a 5-point Likert scale (1 = not at all to 5 = extremely). The measure comprises three subscales, each with two items: intrusion (e.g., “I think about what happened even when I don’t want to”), avoidance (e.g., “I try not to think about it”), and hyperarousal (e.g., “I feel defensive and alert”). In the present sample, internal consistency was good (α = 0.85).

Prolonged Grief Disorder-13 (PG-13; USA [12]: Portuguese version [41]). The PG-13 consists of 13 items that assess prolonged grief symptoms in line with DSM-5 and ICD-11 criteria. Eleven items are rated on a 5-point Likert scale (1 = not at all to 5 = extremely), covering cognitive, emotional, and behavioral aspects of grief (e.g., difficulty accepting the loss, difficulty trusting others, longing, worry, resentment). Two dichotomous items (yes/no) assess temporal criteria. Internal consistency in this study was excellent (α = 0.95).

Guilt and unfinished business. Experiences of guilt and unfinished business were measured with items developed by the authors. Guilt was assessed with the item “I feel responsible for their death,” rated on a 5-point Likert scale (1 = never to 5 = always). Unfinished business was assessed with two items: “I regret choices or actions I made while my family member was alive” and “I regret not having said some things to my family member that would have been important.” These were also rated on a 5-point Likert scale. A total unfinished business score was obtained by summing the two items, with higher scores reflecting greater regret. Internal consistency was acceptable (α = 0.74), and the two items were moderately correlated (rₛ = 0.58, *p* < 0.001). To ensure content validity, item wording was derived from established theoretical descriptions of guilt and unfinished business in bereavement (e.g., unresolved interpersonal or moral concerns following the death of a loved one). Items were reviewed by domain experts in grief research to confirm clarity, relevance, and conceptual coverage prior to data collection. Evidence of temporal stability was also obtained for the single-item guilt measure, showing good test–retest reliability over a three-week interval (ICC(3,1) = 0.69, 95% CI [0.59, 0.77], *p* < 0.001). Convergent validity was supported by moderate correlations between guilt and unfinished business and related constructs, including grief (r_s_ = 0.28–0.32), rumination (r_s_ = 0.35–0.37), and posttraumatic stress symptoms (r_s_ = 0.31–0.35), all ps < 0.001, consistent with theoretical expectations.

Utrecht Grief Rumination Scale (UGRS; The Netherlands [28]; Portuguese version [42]). The UGRS comprises 15 items assessing grief-related rumination, each rated on a 5-point Likert scale (1 = never to 5 = always). Items capture thoughts about negative emotional reactions, unfairness of the death, counterfactual thinking, personal meaning, and consequences of the loss (e.g., “Have you analyzed the personal meaning of the loss for you?”; “Have you thought about decisions that could have changed the outcome?”). Internal consistency was excellent in this sample (α = 0.95).

### 2.4. Statistical Analyses

All analyses were conducted using IBM SPSS Statistics (version 30). Descriptive statistics and Pearson correlations were first computed to examine associations among the main study variables. The magnitude of correlations was interpreted according to Cohen’s [43] criteria: weak (r < 0.30), moderate (r = 0.30–0.49), and strong (r ≥ 0.50). To test the mediating role of rumination in the association between guilt and grief, mediation analyses were performed using PROCESS macro for SPSS (Model 4) [44]. Guilt and unfinished business were entered as independent variables, with prolonged grief symptoms and PTSD symptoms examined as dependent variables in separate models. Rumination was specified as the mediating variable in all models. Indirect effects were estimated using bootstrapping procedures with bias-corrected 95% confidence intervals (CIs). Mediation effects were considered significant if the CI did not include zero. Statistical significance was set at α = 0.05. There were no missing data at either the scale or item level. For each mediation model, both unstandardized and standardized coefficients (β) were reported, along with completely standardized indirect effects (ab_cs), model-level effect sizes (ΔR^2^ and Cohen’s f^2^), and 95% bootstrap CIs based on 5000 samples. The proportion of variance explained (R^2^) was presented for each equation, and the strength of effects was interpreted according to Cohen’s [43] guidelines (small, medium, large). Additionally, based on the empirical benchmarks reported by Fritz and MacKinnon [45], our sample of *N* = 141 provides approximately 80% power to detect small-to-medium indirect effects using the bias-corrected bootstrap test. Because the effects observed in our model fall within the medium-to-large range, the study had sufficient power to detect the indirect effect examined.

## 3. Results

Correlations were performed between the variables under study. In this case, these were guilt, unfinished business, grief, rumination, and PTSD symptoms. All correlations were statistically significant (Table 1).

### Mediation Analyses

**Figure 1:**
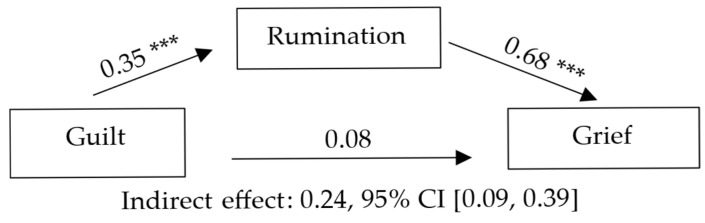
Mediation model illustrating the total mediating effect of rumination between guilt and grief. Note. Standardized coefficients are presented. *p* < 0.001 (***).

Model 1 considered rumination as a mediating variable in the association between guilt and grief. Guilt was associated with higher levels of rumination, which in turn predicted greater grief, indicating a full mediation effect. The overall model explained a substantial proportion of the variance in grief. Detailed statistical results, including standardized coefficients, confidence intervals, and effect sizes, are presented in Table 2 and illustrated in Figure 1.

**Figure 2:**
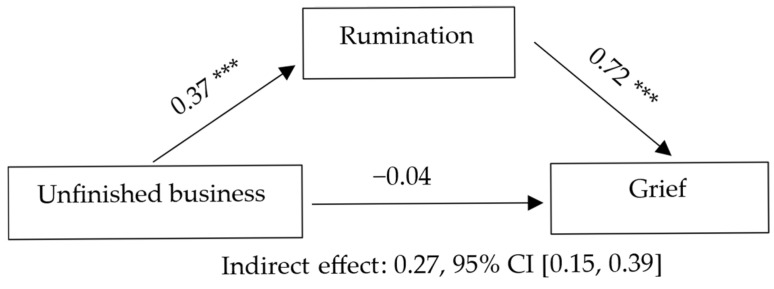
Grief as DV, Unfinished business as IV, and Rumination as mediator. Note. Standardized coefficients are presented. *p* < 0.001 (***).

Model 2 considered rumination as a mediating variable in the association between the unfinished business indicator and grief. Unfinished business was associated with higher levels of rumination, which in turn predicted greater grief, indicating a full mediation effect. The overall model accounted for a substantial proportion of the variance in grief. Detailed statistical results, including standardized coefficients, confidence intervals, and effect sizes, are presented in Table 3 and illustrated in Figure 2.

**Figure 3:**
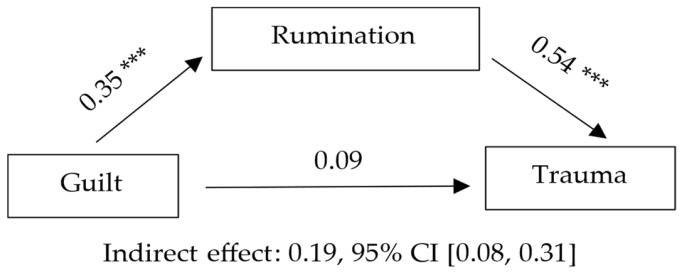
PTSD symptoms as DV, Guilt as IV, and Rumination as mediator. Note. Standardized coefficients are presented. *p* < 0.001 (***).

Model 3 examined the mediating role of rumination in the relationship between guilt and posttraumatic stress symptoms. Guilt was associated with higher levels of rumination, which in turn predicted more severe PTSD symptoms, consistent with a full mediation effect. The overall model explained a meaningful proportion of the variance in PTSD symptoms. Detailed statistical results, including standardized coefficients, confidence intervals, and effect sizes, are presented in Table 4 and illustrated in Figure 3.

**Figure 4:**
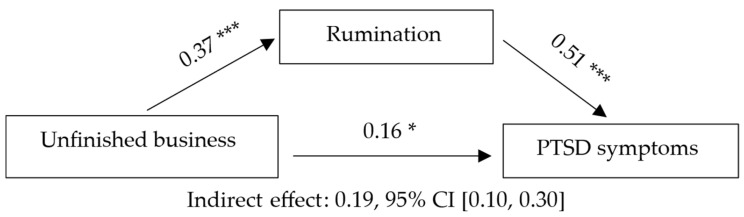
PTSD symptoms as VD, unfinished business as VI, and rumination as mediator. Note. Standardized coefficients are presented. *p* < 0.05 (*); *p* < 0.001 (***).

Model 4 examined the mediating role of rumination in the relationship between unfinished business (IV) and posttraumatic stress symptoms (DV). Unfinished business was associated with higher rumination, which in turn predicted more severe PTSD symptoms. The indirect effect was significant, and, unlike the other models, the direct effect remained significant, indicating partial mediation. Full statistical details (standardized coefficients, confidence intervals, and effect sizes) are reported in Table 5 and depicted in Figure 4.

Across all models, rumination consistently emerged as a significant mediator linking guilt and unfinished business to grief and posttraumatic stress symptoms. The indirect effects were of large magnitude, supported by standardized coefficients, substantial increases in explained variance, and robust bootstrap confidence intervals. These results highlight the central role of ruminative processing in bereavement adjustment, underscoring its relevance as a potential target for clinical intervention and future longitudinal research.

## 4. Discussion

The present study investigated the mediating role of rumination in the associations between guilt, unfinished business, prolonged grief symptoms, and PTSD among bereaved individuals. By integrating cognitive-behavioral perspectives with bereavement research, the study sought to clarify how specific relational, emotional and cognitive experiences contribute to maladaptive adjustment following loss. Although guilt and unresolved relational issues are frequently linked to complicated grief, the mechanisms through which they exert their influence remain insufficiently understood. The findings of this study help illuminate these pathways, demonstrating across four mediation models that rumination plays a central and consistent role in intensifying grief and trauma-related responses after bereavement. These results reinforce a growing body of evidence that emphasizes the primacy of cognitive processing—particularly repetitive negative thinking—over emotional states alone in explaining the persistence of maladaptive grief responses [6].

Across all models, rumination emerged as a robust mediator linking guilt and unfinished business to both prolonged grief and PTSD symptoms. In the first model, guilt did not directly predict prolonged grief symptoms, but its indirect association through rumination was significant. This indicates that guilt may not be inherently harmful; rather, its detrimental effects appear to arise when guilt triggers ruminative, counterfactual thinking that keeps bereaved individuals preoccupied with real or imagined failures (“What should I have done differently?” “If only I had said that”). These findings align with previous work demonstrating that guilt intensifies grief severity when filtered through maladaptive cognitive loops [46]. Although earlier research, such as Wagner et al. [22], identified guilt as a significant predictor of grief, it did not identify the mechanisms underlying that relationship. The current results suggest that guilt increases distress primarily through guilt-related rumination.

A similar pattern emerged with unfinished business. Although unfinished business did not directly predict prolonged grief symptoms, it showed a significant indirect effect through rumination. This finding is consistent with research indicating that relationally unresolved issues—such as unexpressed affection, unresolved conflict, or missed opportunities for connection—are associated with higher levels of grief-related distress [24]. The present study extends this literature by suggesting that the emotional burden generated by unfinished relational issues is maintained not by the issues themselves but by repetitive cognitive engagement with them. Rumination may keep the bereaved mentally attached to aspects of the relationship that remained unfinished—such as unspoken feelings, unresolved conflicts, or missed opportunities for connection or communication, preventing the integration of the loss into autobiographical memory. In this way, unresolved relational concerns prolong grief by perpetuating cognitive immersion in what remained relationally incomplete.

The third mediation model showed that guilt did not directly predict PTSD symptoms but exerted a significant indirect effect via rumination. This aligns with trauma models highlighting rumination as a key mechanism that maintains intrusive memories, self-blame, and physiological arousal [47]. Maladaptive guilt may activate cycles of self-critical thinking and intrusive imagery, thereby sustaining PTSD symptoms. These findings converge with the cognitive model of PTSD proposed by Ehlers and Clark [33], which identifies maladaptive appraisals and repetitive negative thinking as central to the maintenance of trauma-related distress.

In the fourth model, unfinished business again predicted PTSD symptoms indirectly through rumination, and a residual direct effect was retained. This partial mediation suggests that unresolved relational concerns affect trauma-related symptoms through multiple routes. Research among bereaved parents has shown that distress related to unfinished business heightens both grief and trauma responses [48]. Tagay et al. [49] likewise emphasized that rumination around unresolved issues fosters secondary emotions such as regret and longing for validation, thereby intensifying psychological suffering. Together, these findings demonstrate that rumination functions as a stable cognitive process linking both guilt and unfinished business to maladaptive bereavement outcomes.

The present results can be interpreted through several theoretical frameworks. Stroebe and Schut’s [50] Dual Process Model proposes that adaptive adjustment to bereavement requires oscillation between loss-oriented and restoration-oriented coping. Rumination may disrupt this process by overemphasizing loss-oriented processing, keeping attention focused on the deceased and on painful emotions [6], or by serving as an avoidance strategy that prevents individuals from confronting the reality of death [1]. Worden’s [51] four tasks of mourning offer a complementary perspective: if rumination is conceptualized as an avoidance mechanism, it may interfere with all four tasks, including accepting the reality of the loss, processing the pain of grief, adapting to an environment without the deceased, and emotionally relocating the deceased. These frameworks converge in highlighting rumination as a process that obstructs the cognitive and emotional adjustments needed for adaptive bereavement.

A closer examination of the mediation patterns reveals nuances in the absence of direct effects. Most models showed no direct association between guilt or unfinished business and grief or PTSD symptoms, except for unfinished business that retained a direct link to PTSD. The predominance of indirect effects across models underscores the central role of repetitive negative thinking in shaping bereavement outcomes [6,27]. However, it is also possible that the brief measures used for guilt and unfinished business limit the detection of direct effects. These constructs are multidimensional, and single- or two-item indicators may not fully capture the nuances involved. Certain components of guilt and unfinished business, such as self-blame or “if only” thoughts, may overlap conceptually with rumination, thereby channeling their effects through repetitive cognition [1,52]. Future research using more comprehensive measures may help clarify whether these relationships reflect substantive psychological processes or measurement-related constraints.

### 4.1. Clinical Implications

The clinical implications of these findings are significant. Both guilt and unfinished business influence prolonged grief and PTSD symptoms primarily through rumination, making it a crucial therapeutic target in the treatment of bereaved individuals. Assessing the frequency, content, and emotional tone of ruminative thoughts should therefore be a routine part of clinical evaluation. Interventions that directly target repetitive negative thinking appear particularly relevant for clinical practice.

Among the interventions with initial but promising empirical support, exposure-based treatments for complicated grief and behavioral activation have demonstrated reductions in grief-related rumination and avoidance in controlled studies [6,11], although stronger randomized evidence in bereaved samples is still needed. Rumination-Focused Cognitive Behavioral Therapy (RFCBT) stands out as one of the most empirically supported interventions for repetitive negative thinking, with recent meta-analytic findings showing consistent reductions in perseverative counterfactual thinking and abstract, analytical processing [53]. Given that these cognitive features closely resemble grief-specific rumination, RFCBT’s structured functional analysis and its emphasis on concrete, process-oriented thinking make it particularly well suited to the mechanisms identified in the present study. Metacognitive Therapy (MCT) also has a robust evidence base, supported by multiple randomized trials in other clinical populations. Because guilt- and unfinished business–related rumination often emerges from metacognitive beliefs regarding the usefulness or uncontrollability of repetitive thinking, MCT techniques such as detached mindfulness and attentional training may be especially valuable for targeting these mechanisms [54]. Mindfulness-based interventions likewise demonstrate strong empirical support, with randomized controlled trials showing meaningful reductions in rumination and reactivity to intrusive thoughts across disorders. These approaches may assist bereaved individuals by decreasing attentional capture by repetitive thoughts and promoting greater decentering and acceptance [55].

Taken together, these interventions represent promising pathways for targeting the ruminative mechanisms highlighted in this study. Future randomized controlled trials specifically focused on bereavement are needed to clarify their comparative efficacy in reducing grief-related rumination.

More broadly, the findings challenge traditional stage-or task-based models of grief (e.g., Kübler-Ross, Worden). While these models provide helpful frameworks, they may inadequately capture the cognitive processes—such as rumination—that underlie prolonged suffering. A more process-oriented and individualized clinical stance is required, emphasizing flexibility, active listening, emotional validation, and exploration of the mental content associated with loss. Such an approach allows interventions to be tailored to the unique trajectory of each bereaved individual.

At a systemic level, the results highlight the importance of developing specialized grief support programs that incorporate cognitive-focused interventions. Multidisciplinary teams, peer-support groups, and training for health professionals should prioritize the early identification of pathological rumination and the promotion of adaptive strategies. Preventive interventions may be especially valuable during vulnerable periods, such as the first year of bereavement, to reduce the likelihood of prolonged grief or trauma-related disorders.

### 4.2. Limitations and Future Studies

Despite its contributions, this study has several limitations that must be acknowledged. First, the use of a non-probabilistic snowball sampling method limits the generalizability of the results. The sample was predominantly female and highly educated, which may bias the findings and reduce their applicability to other sociodemographic groups. Future research should seek to recruit more diverse and representative samples, taking into account gender, age, socio-economic background, and cultural context.

Second, guilt and unfinished business were measured with brief, author-developed items. Although additional analyses supported their temporal stability and convergent validity, the use of single- and two-item indicators inevitably restricts the assessment of the multidimensional nature of these constructs. This methodological choice was, however, ethically and practically motivated, as the study formed part of a larger longitudinal project involving bereaved participants. To minimize participant burden and potential distress, it was necessary to limit the overall length of the assessment protocol. Future studies should replicate these findings using validated multi-item instruments to further strengthen measurement precision.

Third, although the design was longitudinal, data were collected only at two time points. While this design allows for stronger causal inference than cross-sectional studies, more frequent assessments over extended periods would provide a richer understanding of how rumination evolves dynamically and influences adjustment trajectories over time.

Fourth, the study was conducted during the COVID-19 pandemic, a context that may have profoundly shaped participants’ experiences of loss. Restrictions on in-person farewells, funeral rituals, and social support may have intensified rumination and complicated grieving processes. In addition, the pandemic may have amplified or distorted bereavement trajectories in ways that differ from typical circumstances. Limited access to the deceased, disrupted caregiving roles, and the inability to engage in culturally normative mourning practices may have heightened feelings of helplessness, guilt, and unfinished business, thereby strengthening ruminative tendencies. The prolonged social isolation and reduced availability of informal and formal support systems may also have delayed adaptive emotional adjustment. Consequently, caution is needed when generalizing findings beyond this specific historical context.

Future research should also expand the conceptual scope of inquiry. It will be important to examine the differential roles of guilt and shame, as these emotions, though related, carry distinct implications. Shame, in particular, remains underexplored in bereavement research, despite evidence of its strong association with avoidance and worthlessness [56]. Moreover, studies should explore additional mediators and moderators—such as attachment style, social support, coping strategies, and post-traumatic growth—that may influence how guilt and unfinished business affect bereavement outcomes.

Future research should focus on understanding how rumination impedes healthy adaptation and if these mechanisms can be better explained by the Response Styles Theory [6] or through the Rumination as Avoidance Hypothesis [1].

Methodologically, mixed-methods approaches that integrate qualitative narratives with quantitative data could provide deeper insight into the lived experiences of rumination, guilt, and unresolved issues. Such approaches would allow researchers to capture nuances that standardized instruments may miss.

Also, it is important to consider potential bidirectionality in the observed associations. While the present models conceptualized rumination as the pathway from guilt and unfinished business to grief and PTSD, it is plausible that elevated symptoms also intensify ruminative thinking, creating a self-perpetuating cycle of distress. Moreover, the extremely high correlation between guilt and unfinished business suggests that these constructs may not be entirely distinct and could reflect overlapping dimensions of perceived responsibility and relational incompleteness. Future research should employ more fine-grained measurement strategies, such as cross-lagged panel models (CLPMs) and random-intercept cross-lagged panel models (RI-CLPMs). These would allow for the evaluation of bidirectional pathways and distinguish stable-like differences from dynamic withing-person fluctuations. Such approaches could reveal whether these processes form escalating cycles, maintenance loops or mutually reinforce patterns over time [57,58].

Additionally, cultural norms surrounding death, responsibility, and continuing bonds with the deceased may influence the salience and meaning of guilt and unfinished business, underscoring the importance of cross-cultural validation.

Recent research has highlighted how environmental and contextual disruptions can shape cognitive rumination patterns, including findings on parenting styles [59], exposure to natural versus urban environments [60], and smartphone use [61]. Such disruptions may be particularly relevant in bereavement, as destabilized routines, reduced opportunities for restorative environments, and increased dependence on digital devices can heighten repetitive negative thinking. Given these influences, it is important for future studies to examine how these contextual factors specifically affect grief-related rumination and to explore how such insights may inform the development of targeted interventions.

Finally, future studies should evaluate the effectiveness of targeted interventions and investigate how rumination-based interventions can be applied in the context of bereavement to reduce rumination and facilitate adaptive grieving. Randomized controlled trials would be especially valuable in establishing evidence-based guidelines for clinical practice.

## 5. Conclusions

The present study underscores the central role of grief-specific rumination as a key cognitive mechanism through which guilt and unfinished business shape bereavement-related distress. By demonstrating that these early relational and emotional responses exert their impact primarily through repetitive negative thinking, our findings advance theoretical models that position rumination as a pivotal process in the development and maintenance of prolonged grief and PTSD symptoms. Clinically, these results highlight the importance of incorporating interventions that directly target maladaptive rumination—such as metacognitive, mindfulness-based, or rumination-focused cognitive-behavioral approaches—into bereavement care. Beyond reinforcing the relevance of cognitive pathways, the study points to several directions for future inquiry. Research employing more comprehensive measures of guilt and unfinished business, multi-wave longitudinal designs, and cross-cultural samples is needed to clarify causal dynamics and contextual influences on ruminative processing. Additionally, investigating moderators such as attachment style, social support, and meaning-making may illuminate why some bereaved individuals become trapped in ruminative cycles whereas others adapt more resiliently. Together, these avenues can deepen our understanding of maladaptive grief trajectories and inform more targeted, evidence-based interventions.

## Figures and Tables

**Table 1:** Correlations between the variables under study.

	M (SD)	1	2	3	4
1. Guilt	1.48 (0.90)	-			
2. Unfinished Business	4.88 (2.18)	0.32 ***	-		
3. Grief	25.45 (11.77)	0.28 ***	0.25 **	-	
4. Rumination	39.69 (15.60)	0.36 ***	0.35 ***	0.74 ***	-
5. PTSD	16.35 (5.76)	0.31 ***	0.30 ***	0.82 ***	0.69 ***

Note. ** *p* < 0.01; *** *p* < 0.001.

**Table 2:** Mediation Model of Guilt, Rumination, and Grief: Standardized Path Coefficients and Indirect Effect (Bootstrap 95% CI).

Path/Effect	B	SE	β	95% CI	*p*
Guilt → Rumination (a)	6.12	1.38	0.35	[3.40, 8.85]	<0.001
Rumination → Grief (b)	0.51	0.05	0.68	[0.42, 0.61]	<0.001
Guilt → Grief (total, c)	4.15	1.05	0.32	[2.07, 6.24]	<0.001
Guilt → Grief (direct, c′)	1.02	0.84	0.08	[−0.64, 2.69]	0.225
Indirect (a·b)	3.13	1.08	—	[1.19, 5.41]	—

Note. Letters in parentheses indicate the paths of the mediation model: a = Guilt → Rumination; b = Rumination → Grief; c = total effect; c′ = direct effect; a·b = indirect effect. B = unstandardized coefficient; SE = standard error; β = standardized coefficient; CI = confidence interval. The statistical significance of the indirect effect was determined using a bootstrap confidence interval (5000 samples). Completely standardized indirect effect (ab_cs): 0.24, 95% CI [0.09, 0.39]. Model R^2^ = 0.502, F (2, 138) = 69.63, *p* < 0.001. ΔR^2^ (relative to total effect model) = 0.40; f^2^ = 1.01 (large effect).

**Table 3:** Mediation Model of Unfinished Business, Rumination, and Grief: Standardized Path Coefficients and Indirect Effect (Bootstrap 95% CI).

Path/Effect	B	SE	β	95% CI	*p*
Unfinished business → Rumination (a)	2.66	0.56	0.37	[1.55, 3.77]	<0.001
Rumination → Grief (b)	0.54	0.05	0.72	[0.45, 0.64]	<0.001
Unfinished business → Grief (total, c)	1.24	0.44	0.23	[0.36, 2.12]	0.006
Unfinished business → Grief (direct, c′)	−0.20	0.35	−0.04	[−0.90, 0.49]	0.563
Indirect (a·b)	1.44	0.34	—	[0.80, 2.17]	—

Note. Letters in parentheses indicate the paths of the mediation model: a = Guilt → Rumination; b = Rumination → Grief; c = total effect; c′ = direct effect; a·b = indirect effect. B = unstandardized coefficient; SE = standard error; β = standardized coefficient; CI = confidence interval. Statistical significance of the indirect effect was determined using a bootstrap confidence interval (5000 samples). Completely standardized indirect effect (ab_cs) = 0.27, 95% CI [0.15, 0.39] (bootstrap, 5000 samples). Model statistics: R = 0.71, R^2^ = 0.50, F (2, 138) = 68.49, *p* < 0.001. ΔR^2^ (relative to total effect model) = 0.45; f^2^ = 0.99 (large effect).

**Table 4:** Mediation Model of Guilt, Rumination, and PTSD Symptoms: Standardized Path Coefficients and Indirect Effect (Bootstrap 95% CI).

Path/Effect	B	SE	β	95% CI	*p*
Guilt → Rumination (a)	6.12	1.38	0.35	[3.40, 8.85]	<0.001
Rumination → PTSD symptoms (b)	0.19	0.03	0.54	[0.14, 0.24]	<0.001
Guilt → PTSD symptoms (total, c)	1.69	0.49	0.28	[0.72, 2.67]	<0.001
Guilt → PTSD symptoms (direct, c′)	0.53	0.45	0.09	[−0.35, 1.42]	0.236
Indirect (a·b)	1.16	0.39	—	[0.48, 2.03]	—

Note. Letters in parentheses indicate the paths of the mediation model: a = Guilt → Rumination; b = Rumination → Grief; c = total effect; c′ = direct effect; a·b = indirect effect. B = unstandardized coefficient; SE = standard error; β = standardized coefficient; CI = confidence interval. Statistical significance of the indirect effect was determined using a bootstrap confidence interval (5000 samples). Completely standardized indirect effect (ab_cs) = 0.19, 95% CI [0.08, 0.31] (bootstrap, 5000 samples). Model statistics (DV = PTSD symptoms): R = 0.58, R^2^ = 0.33, F (2, 138) = 34.75, *p* < 0.001. ΔR^2^ (relative to total effect model) = 0.26; f^2^ = 0.50 (large effect).

**Table 5:** Mediation Model of Unfinished Business, Rumination, and PTSD Symptoms: Standardized Path Coefficients and Indirect Effect (Bootstrap 95% CI).

Path/Effect	B	SE	β	95% CI	*p*
Unfinished business → Rumination (a)	2.66	0.56	0.37	[1.55, 3.77]	<0.001
Rumination → PTSD symptoms (b)	0.18	0.03	0.51	[0.13, 0.23]	<0.001
Unfinished business → PTSD symptoms (total, c)	0.88	0.20	0.36	[0.49, 1.28]	<0.001
Unfinished business → PTSD symptoms (direct, c′)	0.41	0.18	0.16	[0.05, 0.77]	0.028
Indirect (a·b)	0.47	0.13	—	[0.24, 0.77]	—

Note. Letters in parentheses indicate the paths of the mediation model: a = Guilt → Rumination; b = Rumination → Grief; c = total effect; c′ = direct effect; a·b = indirect effect. B = unstandardized coefficient; SE = standard error; β = standardized coefficient; CI = confidence interval. Statistical significance of the indirect effect was determined using a bootstrap confidence interval (5000 samples). Completely standardized indirect effect (ab_cs) = 0.19, 95% CI [0.10, 0.30] (bootstrap, 5000 samples). Model statistics (DV = PTSD symptoms): R = 0.59, R^2^ = 0.35, F (2, 138) = 37.39, *p* < 0.001. ΔR^2^ (relative to total effect model) = 0.23; f^2^ = 0.54 (large effect).

## Data Availability

The original contributions presented in this study are included in the article. Further inquiries can be directed to the corresponding author.
